# Subacromial Balloon Implantation for the Treatment of Irreparable Posterosuperior Rotator Cuff Tears

**DOI:** 10.1016/j.eats.2023.03.021

**Published:** 2023-07-10

**Authors:** Erick M. Marigi, Kareme D. Alder, Mark M. Morrey, Joaquin Sanchez-Sotelo

**Affiliations:** Department of Orthopedic Surgery, Mayo Clinic, Rochester, Minnesota, U.S.A.

## Abstract

Despite the development of various therapeutic options, surgical management of irreparable rotator cuff tears (IRCTs) remains controversial. Recently, implantation of a biodegradable subacromial balloon spacer (InSpace, Stryker Corporation; Kalamazoo, MI) has gained considerable interest for the treatment of certain IRCTs. The reported outcome of balloon implantation has not been consistent, likely due to differing indications and technical approaches. The purpose of this article is to present a reproducible arthroscopic technique for implantation of a subacromial balloon and to review the literature published to date, regarding the efficacy and outcomes of this procedure.

## Introduction

The term functionally irreparable rotator cuff tear (FIRCT) refers to those rotator cuff tears that cannot be repaired primarily or are doomed to failure if repair is attempted.^1^, ^2^, ^3^ Some factors are known to correlate with increased risk of failure include large tear size, chronicity of the injury, length of the remaining torn tendon, and fatty infiltration of the rotator cuff musculature.^1^, ^2^, ^3^ Despite the prevalence and increasing understanding of rotator cuff pathology, controversy exists over the optimal for FIRCTs.

Proposed management strategies are often the result of shared decision-making based on the functional status of each individual shoulder, as well as the nature of the tear and surgeon preferences.^2^ Nonoperative modalities, such as nonsteroidal anti-inflammatory drugs and physical therapy are usually considered first, especially in elderly patients. Surgical interventions for IRCTs range from arthroscopic RC debridement with biceps tenotomy to partial rotator cuff repair with or without augmentation, tendon transfers, superior capsular reconstruction (SCR), and reverse total shoulder arthroplasty (RSA).^4^, ^5^, ^6^, ^7^, ^8^, ^9^, ^10^

More recently, another option, an implantable biodegradable subacromial balloon spacer (InSpace, Stryker Corporation; Kalamazoo, MI) has become available.^11^ First used in 2012, the subacromial balloon spacer was developed as a novel treatment modality for certain IRCTs.^11^^,^^12^ The purpose of this article is to present a reproducible arthroscopic technique for implantation of the subacromial balloon spacer and to review the literature regarding the efficacy and outcomes of this procedure.

Ethics approval and consent to participate were obtained in this study by the Mayo Clinic (Institutional Review Board # 21-006049).

### Patient Selection

**Fig 1:**
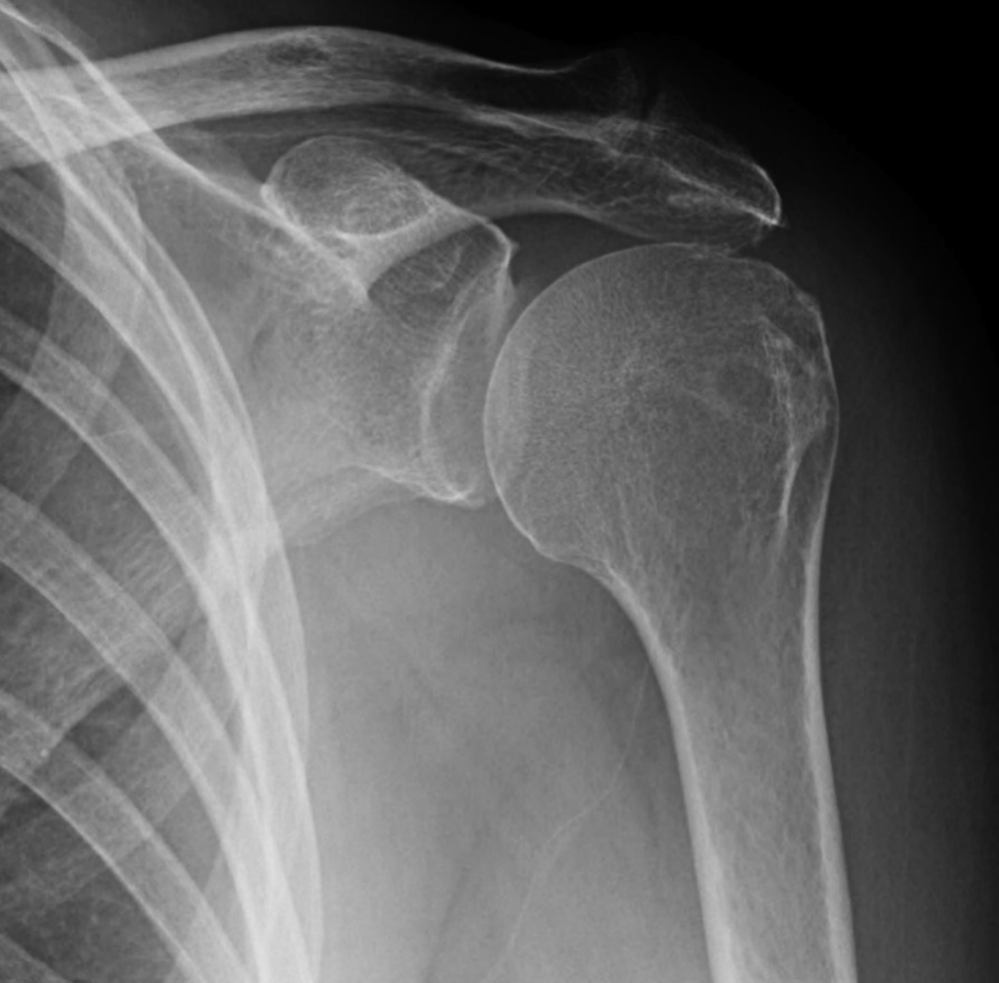
Preoperative anteroposterior radiograph of the shoulder.

**Fig 2:**
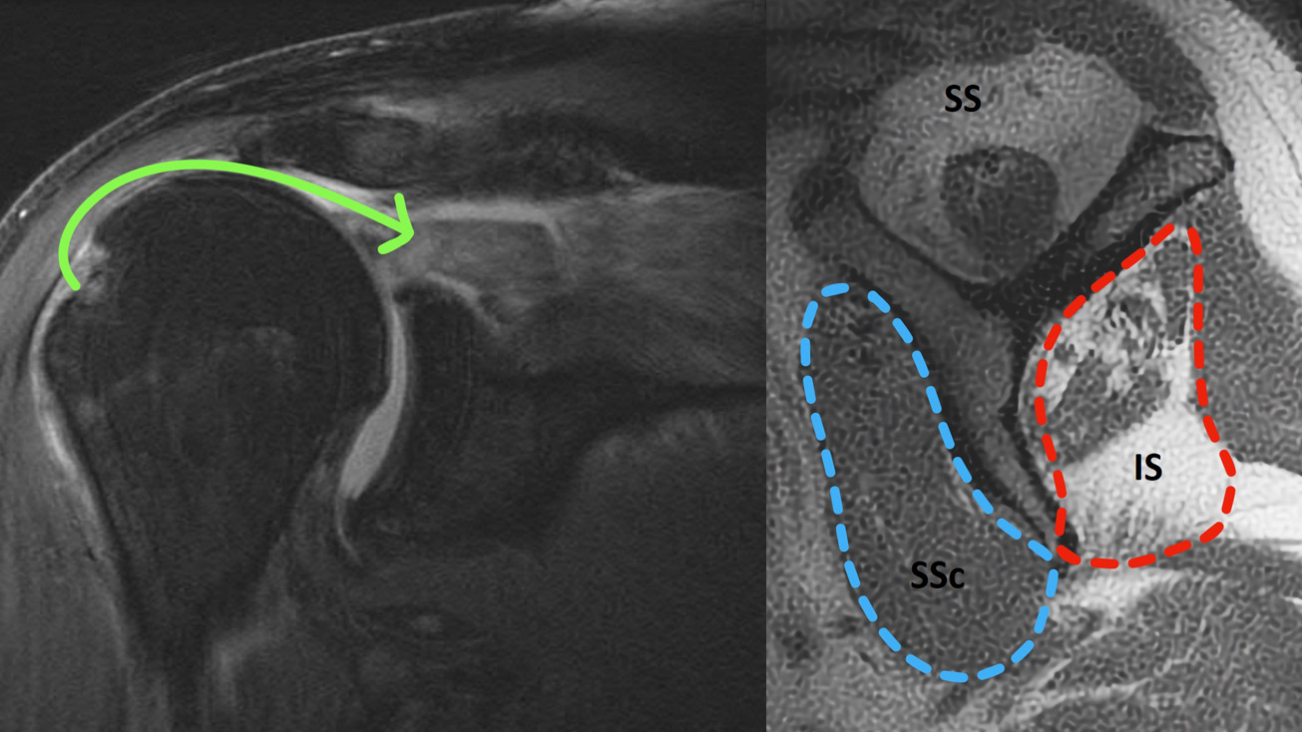
Preoperative coronal magnetic resonance imaging (MRI) demonstrating a retracted rotator cuff tear (arrow). Sagittal MRI demonstrating atrophy of the supraspinatus (SS) and infraspinatus (IS) muscles. SSc, subscapularis.

During evaluation of all patients, a thorough history and physical examination of the afflicted shoulder is performed. The timeframe and chronicity of the rotator cuff injury and subsequent symptoms are noted. The patient's current functional abilities and limitations are also observed. Assessment of pain and the level of concomitant glenohumeral joint (GHJ) arthritis are factored. Standard imaging of the shoulder, including plain films with anteroposterior, lateral, and axillary views, are acquired to assess for the presence of arthritic changes or humeral head or glenoid deformity that can occur with profession of rotator cuff disease (Fig 1). Magnetic resonance imaging is performed to visualize and characterize the rotator cuff, its muscles, and the condition of the articular cartilage and other structures (Fig 2).

In the United States, the Food and Drug Administration has approved implantation of a subacromial balloon for patients with an irreparable posterosuperior rotator cuff tear who are 65 years old or older. The reported outcomes are best in patients with minimal to no glenohumeral joint arthritis (Hamada grade 1 or 2),^13^ a completely intact subscapularis, adequate preoperative active motion, and no external rotation lag sign.

Certain surgeons consider implantation of a subacromial balloon in patients younger than 65 years of age, or when an associated subscapularis tear is present but can be repaired. Another reported use of the subacromial balloon is to augment a primary repair in circumstances when the repair is tenuous; theoretically, the implanted balloon would 1) provide uniformly increased contact pressure between the repaired posterosuperior cuff and the greater tuberosity, and 2) maintain the humeral head centered in reference to the glenoid, decreasing any stress the repair might suffer with a residual tendency of the humeral head to migrate inferiorly. Overall indications and contraindications are summarized in Table 1.

**Table 1:** Indications and Contraindications for Implantation of a Subacromial Balloon

*Indications*
• Irreparable posterosuperior rotator cuff tear • Intact or reparable subscapularis • Preserved active and passive shoulder motion • Limited or no evidence of osteoarthritis • No external rotation lag sign • Age over 65 for Food and Drug Administration, on-label application
**Contraindications**
• Pseudo-paralysis or severe restriction of motion of the glenohumeral joint • Irreparable subscapularis tear • Advanced glenohumeral joint cartilage degeneration • Allergy to poly-l-lactide-co-ϵ-capro-lactone or related synthetic copolymers • Active glenohumeral or systemic infection

## Surgical Technique

### Patient Positioning

The procedure is typically performed under general anesthesia with or without use of an interscalene block. The patient may be placed in the beach chair or lateral decubitus position at the preference of the surgeon; in our practice, we prefer the beach chair position. The surgical field is prepared and draped with standard sterile techniques. Use of a dedicated pneumatic arm holder for arthroscopic surgery is very helpful (Tenet T-Max Beach Chair and Spider arm positioner; Smith & Nephew, Memphis, TN).

### Arthroscopic Assessment and Additional Procedures

The arthroscopic camera (Stryker 4.0-mm Precision Ideal Eyes HD Autoclave Arthroscope, C-Mount, Speed-Lock (Stryker Corporation, Kalamazoo, MI) may be inserted through either a standard posterior glenohumeral portal or a more lateral and superior posterolateral portal at the preference of the surgeon. In the presence of an irreparable posterosuperior cuff tear, it is typically more effective to start the procedure with the camera in the posterolateral subacromial portal.

If the arthroscopic assessment reveals an unexpected irreparable subscapularis tear or advanced glenohumeral joint arthritis, implantation of the subacromial balloon should probably be abandoned. A repairable subscapularis tear is considered by some a relative contraindication for balloon implantation, but in our practice, we do move forward with implantation of a subacromial balloon if a repairable subscapularis tear is encountered and can be properly repaired. When indicated, a resection of the lateral end of an arthritic acromioclavicular joint or biceps tenotomy or tenodesis are performed as well, although some surgeons strongly favor tenotomy over tenodesis for balloon cases, so that additional protection of the shoulder after surgery is not needed.

A lateral subacromial portal is then established unless it was already created for biceps surgery or subscapularis repair. Excessive debridement of the bursa and tissue at the subacromial space should be avoided since postoperative migration of the balloon is more likely to occur if all anterior and posterior subacromial tissue is removed. The subacromial debridement is typically limited to the minimum amount of tissue removal required for adequate visualization.

**Fig 3:**
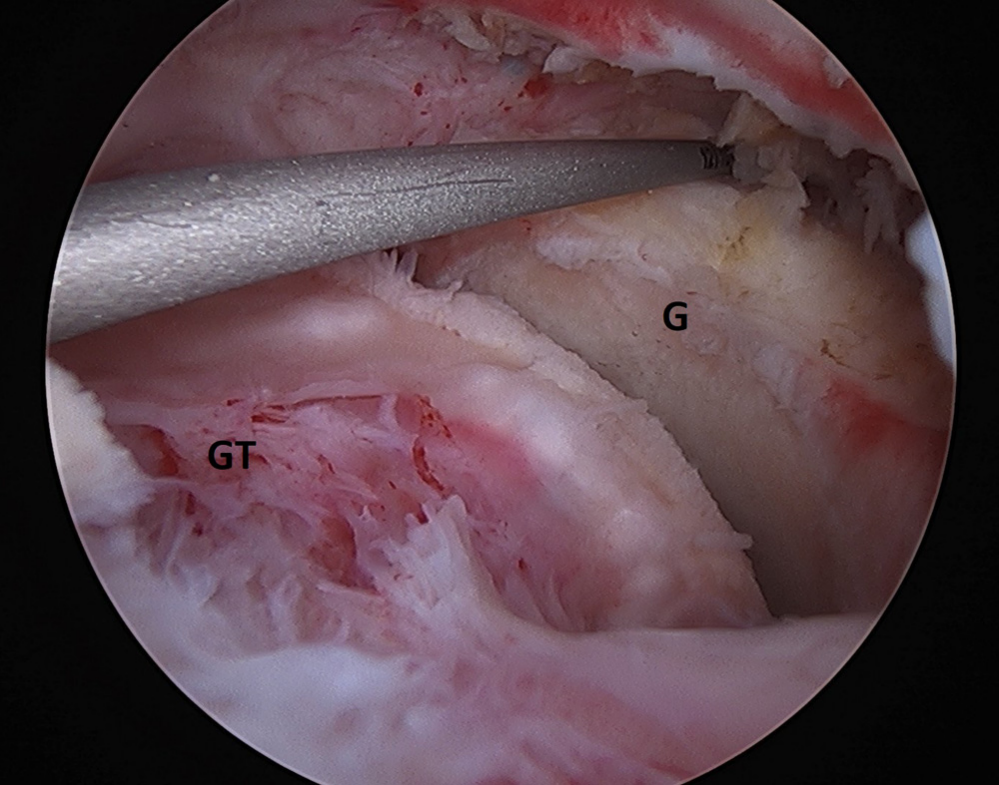
Arthroscopic picture of a right shoulder viewing from a posterior portal with a lateral working portal. Measurement of the footprint defect starting 1 cm medial to the superior glenoid (G) rim to the greater tuberosity (GT).

The next step of the procedure is aimed to determine the size of the balloon to be implanted. A commercially available measurement device may be inserted through the lateral subacromial portal. It is generally recommended that the balloon overlaps with the superior glenoid by 1 cm and extends laterally to the location of the greater tuberosity. Alternatively, a standard arthroscopic probe (Arthrex, Naples, FL) can be used to estimate that distance between 1 cm medial to the glenoid face and the lateral aspect of the greater tuberosity (Fig 3). The balloon is manufactured in 3 sizes, and the size is selected on the basis of this measurement (Table 2). If the surgeon elects to use the balloon to neutralize or augment a tenuous or partial rotator cuff repair, these measurements should be completed before the cuff repair.

**Table 2:** Balloon Sizing and Inflation Chart

Measured *D*istance	Size	Maximum *I*nflation *V*olume	Recommended *F*inal *V*olume
<40 mm	Small	15–17 cc	9–11 cc
40–50 mm	Medium	22–24 cc	16–16 cc
>50 mm	Large	40 cc	23–25 cc

### Subacromial Balloon Setup and Implantation

**Fig 4:**
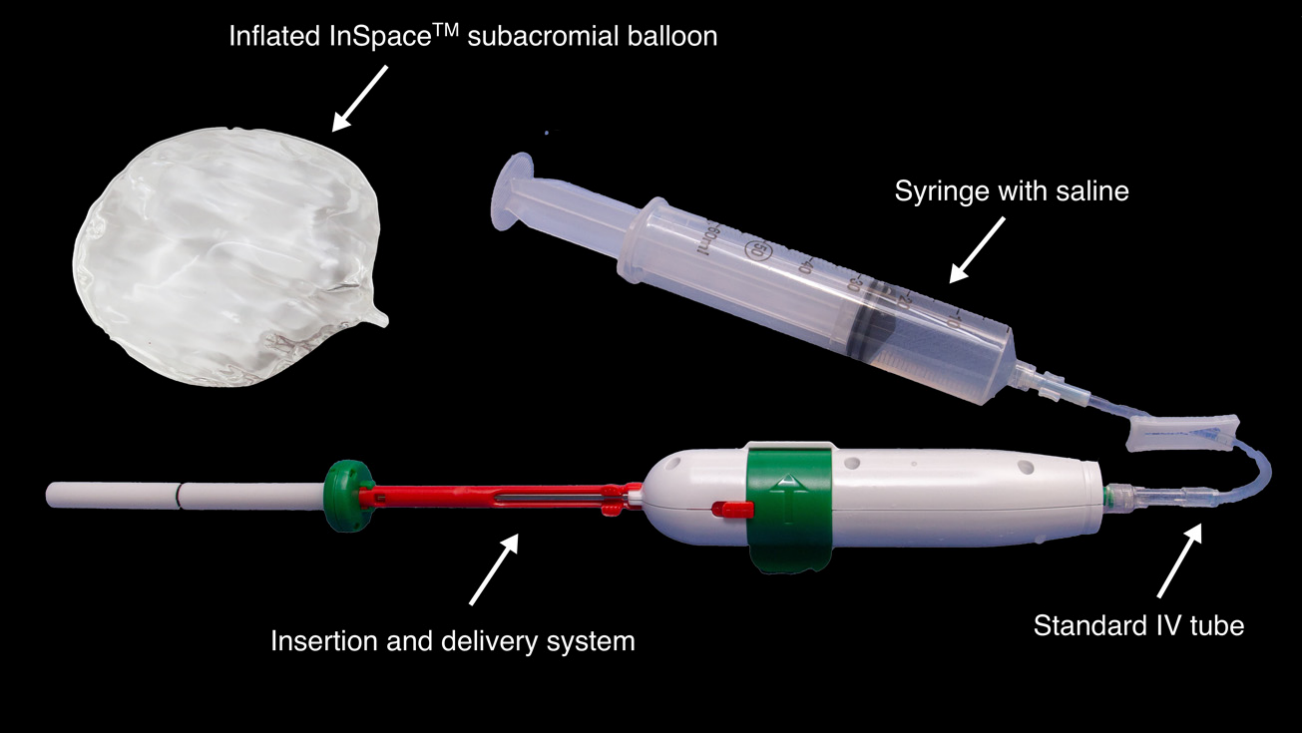
Subacromial balloon and the associated delivery system. The device is connected to a standard intravenous tube and syringe with saline.

Placement of the subacromial balloon (InSpace, Stryker Corporation; Kalamazoo, MI) is typically placed from the lateral portal, while visualizing from the posterior portal. The balloon comes folded inside a dedicated delivery system. A large syringe and an intravenous tube are opened, as well as under sterile conditions. The subacromial balloon is first prepared by filling the sterile syringe with warmed saline. The volume of saline is selected, according to the size of the balloon selected (Table 2). The syringe is slightly overfilled, so that once the syringe is connected to the intravenous tube, all air bubbles are meticulously removed from the syringe and tube prior to connection to the delivery system (Fig 4).

**Fig 5:**
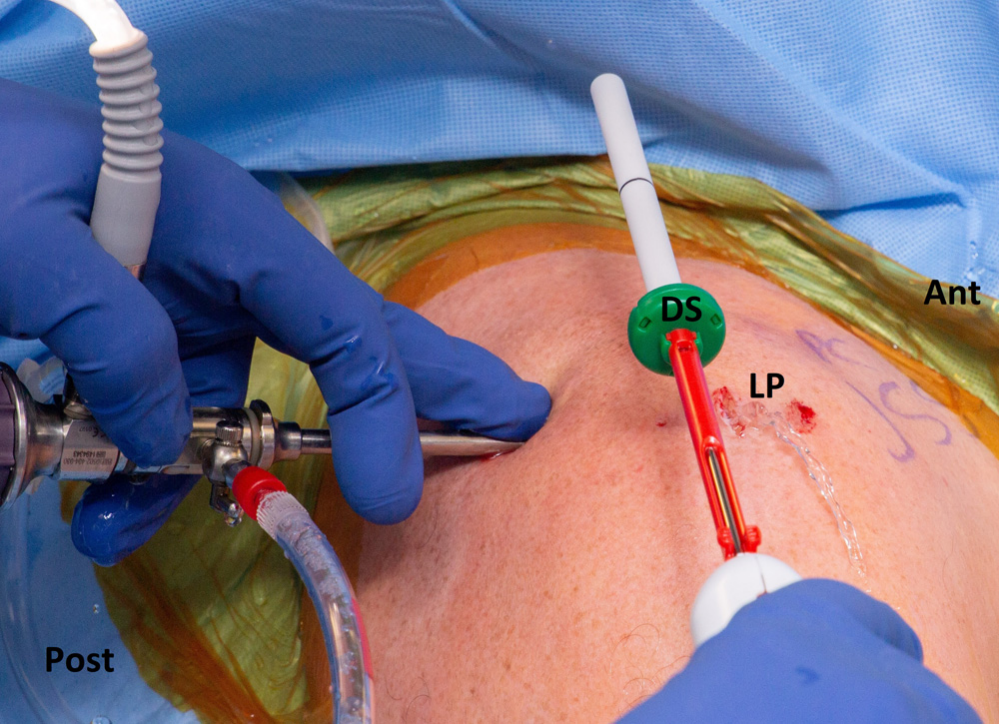
Right shoulder in beach chair position showing the camera in the posterior viewing portal. Insertion of the subacromial balloon delivery system (DS) is made into the subacromial space through the lateral portal (LP) protected by an outer sheath. Ant, anterior; Post, posterior.

**Fig 6:**
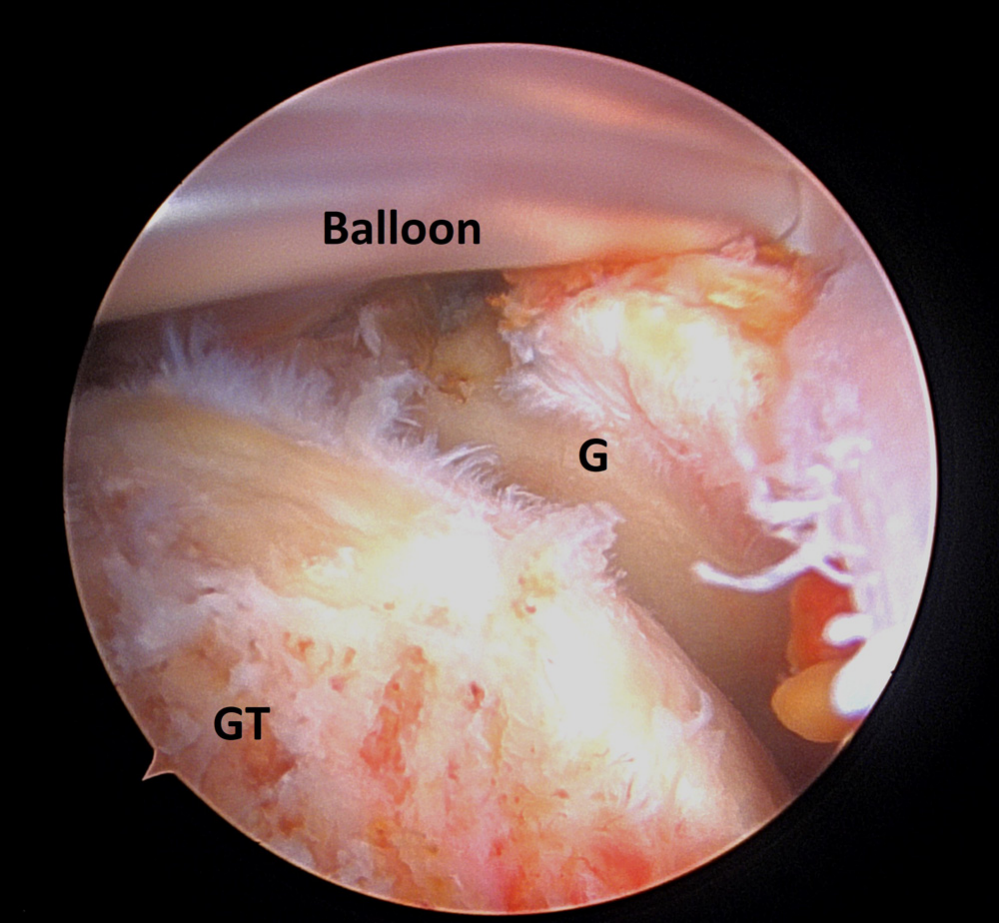
Arthroscopic picture of a right shoulder viewing from a posterior portal. Subacromial balloon is inserted at the desired location, above the glenoid rim (G) and greater tuberosity (GT).

**Fig 7:**
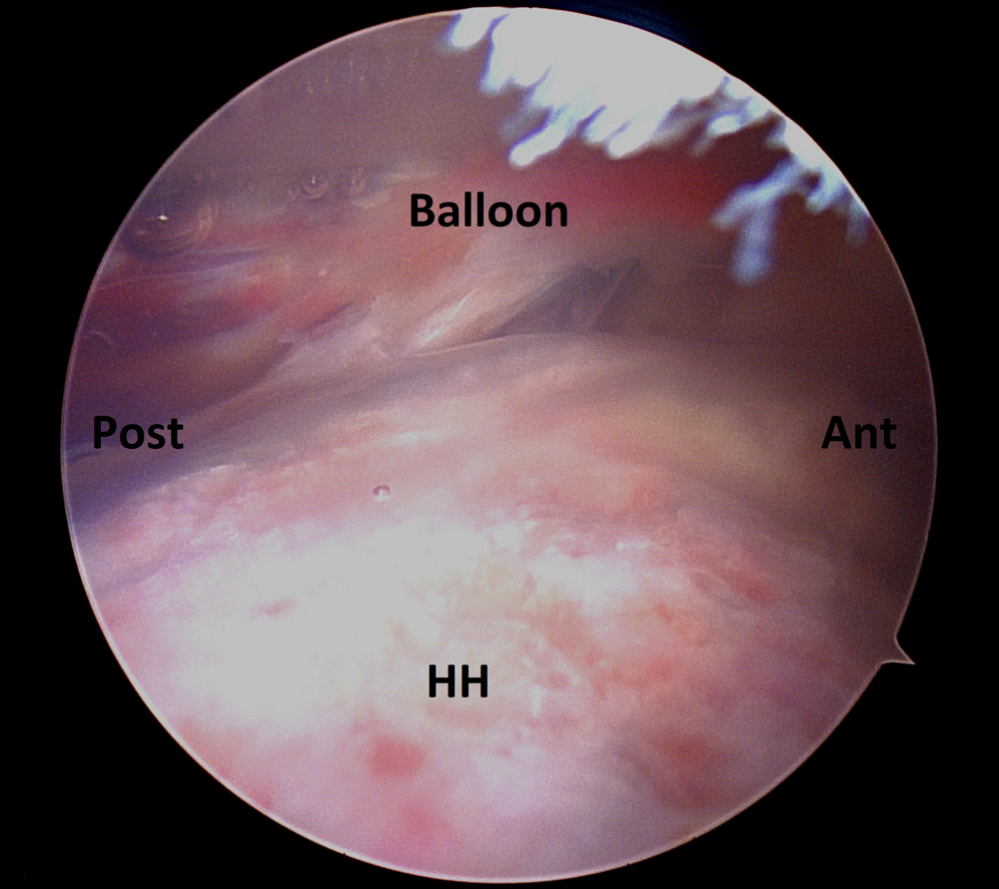
Arthroscopic picture of a right shoulder viewing from a lateral subacromial portal. Balloon is then inflated with sterile saline to the desired volume. Ant, anterior; Post, posterior; HH, humeral head.

Prior to insertion of the delivery system, care must be taken to ensure that the skin incision for the lateral portal is long enough and that bursal tissue underneath the lateral deltoid does not impede advancement of the delivery system (Fig 5). The canula that harbors the balloon is inserted until ∼1 cm of canula overlaps with the superior aspect of the glenoid, and the dark marker line on the canula is at the level of the lateral aspect of the greater tuberosity (Figs 6 and 7).

**Fig 8:**
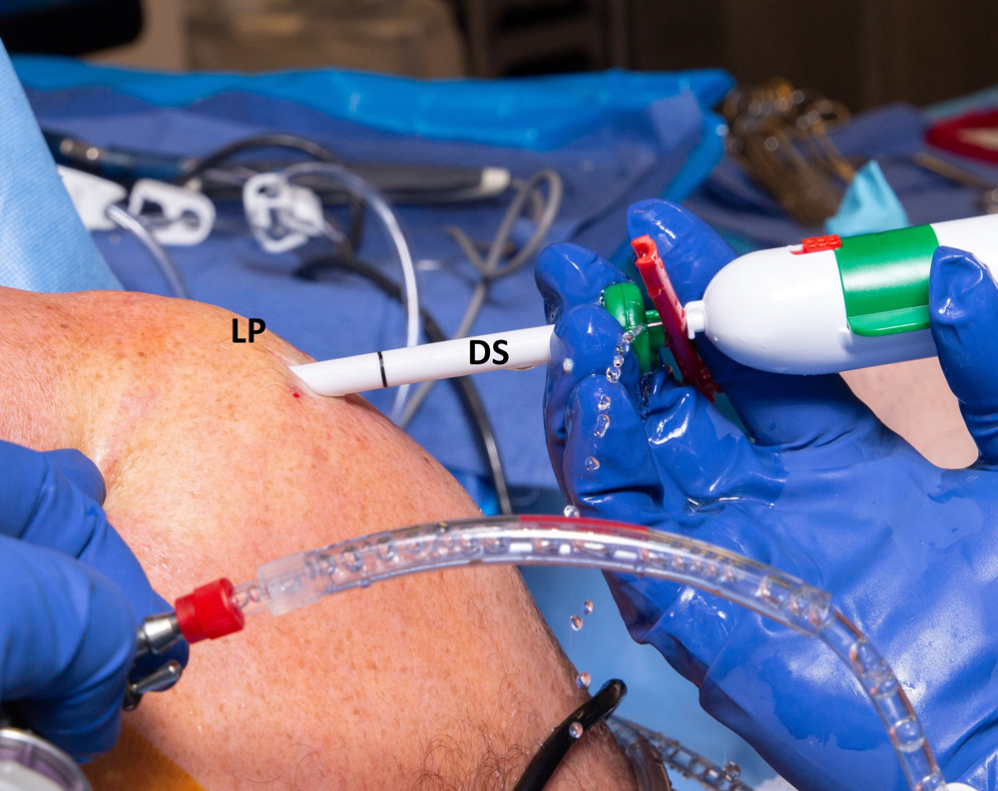
Right shoulder in beach chair position, with the delivery system (DS) in the lateral subacromial portal (LP). The balloon is then sealed using the internal mechanism, and the inserter is removed.

Once the surgeon is satisfied with the anticipated position of the device, the safety mechanism of the delivery system is unlocked, and the protective canula is backed out, taking care not to change the position of the balloon. Once the balloon is completely uncovered by the cannula (Fig 8), all saline in the syringe is advanced into the balloon to fully expand it and remove all wrinkles, and then a small amount of fluid is retrieved to leave the balloon inflated with the right amount of fluid, according to published guidelines (Table 2).^14^ The IV tube is then closed, and the corking mechanism of the balloon is used to seal the balloon. The delivery system is then discarded (see surgical technique Video 1).

### Final Evaluation, Closure, and Rehabilitation

Once insertion of the balloon is completed, some surgeons move the shoulder through a range of motion to confirm that the balloon remains stable. If the balloon is easily dislodged, a spinal needle may be introduced through a portal to lance the inserted balloon, so that another may be placed. If the balloon is stable within the subacromial space, all instrumentation may be removed, and the portal holes are closed in the standard fashion. Diagnostic radiographs may be taken immediately after surgery or at the first postoperative visit to assess radiographic restoration of the acromiohumeral interval. The shoulder is placed in a postoperative sling.

### Postoperative Protocol

The ideal postoperative protocol after implantation of a subacromial balloon has not been fully elucidated. In one prospective randomized study comparing balloon implantation with partial cuff repair, all patients assigned to balloon implantation underwent a rotator cuff repair protocol, with 6 weeks of immobilization followed by range of motion exercises and eventually elastic band strengthening.^15^

When implantation of the subacromial balloon is performed largely as an isolated procedure (maybe with a biceps tenotomy or distal clavicle resection), without biceps tenodesis or repair of a subscapularis tear, some surgeons prefer a faster rehabilitation protocol, maintaining the shoulder in a sling for only 2 weeks and initiating formal rehabilitation between 2 and 4 weeks after the procedure. The benefit of a faster rehabilitation protocol must be balanced against the potential for more frequent balloon migration with early range of motion (Tables 3 and 4).

**Table 3:** Key Pearls and Pitfalls of the Technique

*Pearls*
• Perform a thorough clinical and arthroscopic evaluation of the subscapularis to ensure an intact tendon or reparable subscapularis tear. • Perform a limited subacromial bursectomy from the glenoid rim to the greater tuberosity to allow for appropriate visualization and sizing of the balloon. • If measurement of the balloon is between two sizes, consider using the smaller spacer to limit excessive distention of the subacromial space.
Pitfalls
• Avoid excessive removal of the medial subacromial bursa and division of the coracoacromial ligament, as this may lead to medial migration of the balloon. • Ensure optimal inflation of the balloon, according to the recommended final volume for the size of the balloon. Underinflation increases the risk of migration and overinflation may place excessive tension on the deltoid.

**Table 4:** Advantages and Limitations of the Technique

*Advantages*
• Less invasive, lower risk procedure with a relatively short operative time • Quicker time to postoperative pain relief and a faster rehabilitation potential • Preserves native shoulder anatomy allowing for an easier transition to revision treatment options in the event of failure
Limitations
• Ideal procedure for a small select group of patients with an irreparable rotator cuff tear. • Contraindicated in those with an allergy to poly-l-lactide-co-ϵ-capro-lactone or related synthetic copolymers • Biodegradable, likely representing a temporary solution • Long-term data are lacking, and some short-term research studies have reported contradictory results.

## Discussion

Although many successful innovations and technical advances in shoulder surgery have been made, management strategies regarding irreparable rotator cuff tears continue to generate controversy and debate among surgeons. Currently, both clinical practice guidelines and the available literature recommend treatment decisions to be made as a shared decision-making process between the patient and surgeon, taking into account individual patient shoulder pathology, expectations, and surgeon experience.^3^^,^^16^ In this article, we describe in detail patient selection factors and a reproducible arthroscopic technique for implantation of a subacromial balloon spacer, which has the potential to serve as one useful treatment modality for the management of FIRCTs. Notable technical points include adequate visualization, selectively addressing associated pathology, preparation of the subacromial space to create a contained pocket for the implant, and size and delivery selection, according to published guidelines.

Implantation of the subacromial balloon spacer presents a novel technique for the treatment of massive irreparable rotator cuff tears. The implant was designed to be a minimally invasive method that helps promote normal shoulder biomechanics by preventing proximal migration of the humeral head, thus improving the ability of the deltoid to actively elevate the arm.^11^^,^^17^ Biomechanically, Lobao et al.^18^ demonstrated that the subacromial balloon did, indeed, re-establish native glenohumeral joint (GHJ) contact pressures and lowered high-riding humeral heads into a more native position, thus increasing the efficacy of the deltoid's action and normalizing the pathologic changes found in rotator cuff arthropathy. Furthermore, Chevalier et al.^19^ performed a cadaveric study showing that implantation of the subacromial balloon decreased peak pressures in the GHJ and increased subacromial space load distribution. The authors suggested that these alterations in the GHJ decreased stress on the rotator cuff and could also prevent or limit further development of rotator cuff pathology.^19^ An additional benefit of the system is that the implant may be easily and quickly implanted arthroscopically, which allows for concomitant arthroscopic procedures, such as tendon repair or debridement to be performed at the time of balloon introduction.^20^

Clinically, subacromial balloon spacer implantation has been suggested as a useful method for pain relief and improved function in patients with FIRCTs.^12^^,^^21^ In a prospective study by Senekovic et al.,^22^ implantation of the subacromial balloon demonstrated improvements in pain, activities of daily living, strength, and constant scores from 33.4 to 65.4 points at 3 years of follow-up. Other investigations have also observed that the subacromial balloon improves the range of motion of those with IRCTs in isolation.^23^^,^^24^ However, Holschen et al.^25^ performed a retrospective case control study of 23 patients comparing arthroscopic debridement and partial repair with a balloon spacer and without a balloon spacer. Although the authors observed improvements in both groups, they also detected a higher absolute improvement in the balloon group supporting consideration of balloon use in combination with other RC procedures.

Although the InSpace subacromial balloon spacer is expected to degrade within 12 months of implantation, studies have published clinical improvements outlasting this timeframe. Verma et al.^15^ performed a randomized study comparing 93 patients with a balloon spacer to 91 partial rotator cuff repair with significant clinical improvements in ASES scores up to 2 years out. However, the balloon cohort also experienced a faster recovery, with significantly greater forward elevation at all time points up to 2 years. Similarly, Piekaar et al.^26^ showed continued improvements in pain relief and Oxford shoulder scores at 3-year follow-up. Likewise, Senekovic et al.^27^ showed sustained improvements in Constant scores at 3 and 5 years postoperatively. Szabo hypothesized that these findings may be due to scarring of the subacromial space that occurs after implantation, which subsequently acts similarly to a superior capsule reconstruction.^28^ At the present moment, this remains unconfirmed and represents an ongoing area of investigation.

Interestingly, given the sustained improvements superseding the estimated lifespan of the implant, the subacromial balloon spacer has been viewed as potentially the most cost-effective treatment modality for IRCTs.^29^ Castagna et al.^29^ attempted to quantify this using an expected-value decision analysis model based on Medicare and published literature costs to compare a subacromial balloon, RSA, partial RCR, and PT. Their findings suggested that subacromial balloon implantation was the lowest cost surgical option and the most beneficial with respect to patient and economic outcomes. Although this provided some insight on relative costs between certain treatment modalities of IRCTs, it must be noted that medical treatment is often customized to the individual patient, and economic analyses often limit the applicability to daily clinical practices.^1^^,^^30^

While these early investigations have proposed restoration of natural glenohumeral biomechanics, decreased pain, improved shoulder function, and sustained clinical benefit past the expected time of balloon degradation, additional studies are still needed to determine the full utility of the procedure. The current literature lacks long-term studies of the efficacy of the implant in improving these metrics. Metcalfe et al.^31^ recently published a double-blind multicenter RCT study, in which they observed no difference between the subacromial balloon and RC debridement at 1 year, further questioning the utility of the balloon. However, within their study, most patients had less than 100° of preoperative FE, and subscapularis tears were present in ∼20% of the shoulders and rarely repaired, which surely influenced these results. Regardless, there also remains minimal reporting of implant-related complications or studies demonstrating less favorable outcomes.^32^^,^^33^ Prat et al.^33^ reported a 46% satisfaction rate and a 16.7% complication rate (anterior migration of the balloon, transient deficit of the lateral cutaneous nerve of the forearm, and infection) in 24 shoulders at a mean follow-up of 14 months. Subsequently, Ruiz Iban et al.^32^ also observed a high rate of dissatisfaction with one-third of the patients requiring conversion to RSA and 60% of the remaining patients experiencing an improvement of the constant score >10 points. As such, they concluded that only 40% of patients appeared to benefit from subacromial balloon spacer implantation. As such, continued investigation is needed to help clearly delineate the ideal indications and best conditions for use of the procedure.

At this time, we believe that this subacromial balloon spacer may represent a useful technique in management of IRCTs, but patient selection is critical. This implant may be considered in isolation or as augmentation to a primary RCR, where there is a heightened concern for failure. Technical pearls include performing the procedure in patients without existing arthritis and preserved active and passive range of motion, with a goal for pain improvement. The subscapularis tendon must also be thoroughly evaluated to ensure that it is intact or can be successfully repaired. This allows for the presence of anterior-posterior coupling forces, which are critical for the native shoulder and subacromial balloon's biomechanical function. Additionally, good visualization must be maintained throughout the procedure. As such, performing a limited bursectomy while maintaining hemostasis is essential. Surgeons should avoid excessive removal of soft tissue medial to the superior glenoid rim, as well as anteriorly and posteriorly, to prevent medial migration of the spacer. At the same time, the preparation of the subacromial space should be performed to create a contained pocket for the balloon. Finally, time should be taken to ensure adequate measurement of the subacromial space to guide appropriate sizing of the balloon.

### Conclusion

Management of irreparable rotator cuff tears continues to present challenges despite technical advances and innovation in shoulder surgery. As it stands, there is no clear consensus on a superior operative intervention for all patients. Implantation of a biodegradable subacromial balloon spacer represents a novel technique with the potential to improve pain and function in patients with IRCT by restoring native GHJ biomechanics. This article presents a review of the current literature and our preferred technique for arthroscopic implantation of a subacromial balloon spacer. Future investigations should continue to research the ideal set of indications for this technique, the possibility of device-related complications, and the long-term clinical outcomes of implantation.

## Supplementary Data

ICMJE author disclosure forms

Video 1 Detailed surgical steps of the technique described with a voiceover. The video demonstrates an arthroscopic technique for implantation of a subacromial balloon spacer (InSpace, Stryker Corporation, Kalamazoo, MI). The patient is in the beach chair position with a right-sided operative shoulder. The primary viewing portal is posterior, and the main working portal is lateral. After a routine diagnostic arthroscopy, the subacromial space is prepared with an arthroscopic shaver. The balloon is then sized and implanted through the lateral portal under direct visualization from the posterior portal. Finally, the subacromial balloon spacer (InSpace, Stryker Corporation) system is again highlighted.
